# FN14 and GRP94 expression are prognostic/predictive biomarkers of brain metastasis outcome that open up new therapeutic strategies

**DOI:** 10.18632/oncotarget.5471

**Published:** 2015-10-19

**Authors:** Antonio Martínez-Aranda, Vanessa Hernández, Emre Guney, Laia Muixí, Ruben Foj, Núria Baixeras, Daniel Cuadras, Víctor Moreno, Ander Urruticoechea, Miguel Gil, Baldo Oliva, Ferran Moreno, Eva González-Suarez, Noemí Vidal, Xavier Andreu, Miquel A. Seguí, Rosa Ballester, Eva Castella, Angels Sierra

**Affiliations:** ^1^ Biological Clues of the Invasive and Metastatic Phenotype Group, Molecular Oncology Department, Bellvitge Biomedical Research Institute (IDIBELL), 08907 L'Hospitalet de Llobregat, Barcelona, Spain; ^2^ Universitat Autònoma de Barcelona (UAB), Biochemistry and Molecular Biology Department, Faculty of Biosciences, Campus Bellaterra, Edifici C, Cerdanyola del Vallés, 08193 Barcelona, Spain; ^3^ Structural Bioinformatics Laboratory, Experimental Sciences Department, Universitat Pompeu Fabra-IMIM, Barcelona Research Park of Biomedicine, 08003 Barcelona, Spain; ^4^ Servei d'Anatomia Patològica, Hospital Universitari de Bellvitge, 08907 L'Hospitalet de Llobregat, Barcelona, Spain; ^5^ Biomarkers and Susceptibility Unit, Institut Català d'Oncologia - IDIBELL, Hospital Duran i Reynals, 08907 L'Hospitalet de Llobregat, Barcelona, Spain; ^6^ Breast Cancer Unit and Neuroncology Unit, Institut Català d'Oncologia - IDIBELL, Hospital Duran i Reynals, 08907 L'Hospitalet de Llobregat, Barcelona, Spain; ^7^ Oncology Service, Institut Català d'Oncologia - IDIBELL, Hospital Duran i Reynals, 08907 L'Hospitalet de Llobregat, Barcelona, Spain; ^8^ Radiation Oncology Service, Institut Català d'Oncologia - IDIBELL, Hospital Duran i Reynals, 08907 L'Hospitalet de Llobregat, Barcelona, Spain; ^9^ Transformation and Metastasis Grup, Cancer Epigenetics and Biology Department, IDIBELL, 08907 L'Hospitalet de Llobregat, Barcelona, Spain; ^10^ Pathology Service, Corporació Sanitaria Parc Taulí, 08208 Sabadell, Spain; ^11^ Oncology Service, Corporació Sanitaria Parc Taulí, 08208 Sabadell, Spain; ^12^ Radiation Oncology Service, Institut Català d'Oncologia, Hospital Universitari Germans Trias i Pujol, 08916 Badalona, Spain; ^13^ Pathology Service, Institut Català d'Oncologia, Hospital Universitari Germans Trias i Pujol, 08916 Badalona, Spain; ^14^ Molecular and Translational Oncology Laboratory, Biomedical Research Center CELLEX-CRBC Institut d'Investigacions Biomèdiques August Pi i Sunyer-IDIBAPS 08036 Barcelona, Spain

**Keywords:** biomarkers, brain metastasis, breast cancer, FN14, GRP94

## Abstract

Brain metastasis is a devastating problem in patients with breast, lung and melanoma tumors. GRP94 and FN14 are predictive biomarkers over-expressed in primary breast carcinomas that metastasized in brain. To further validate these brain metastasis biomarkers, we performed a multicenter study including 318 patients with breast carcinomas. Among these patients, there were 138 patients with metastasis, of whom 84 had brain metastasis. The likelihood of developing brain metastasis increased by 5.24-fold (95%CI 2.83–9.71) and 2.55- (95%CI 1.52–4.3) in the presence of FN14 and GRP94, respectively. Moreover, FN14 was more sensitive than ErbB2 (38.27 *vs*. 24.68) with similar specificity (89.43 *vs*. 89.55) to predict brain metastasis and had identical prognostic value than triple negative patients (*p* < 0.0001). Furthermore, we used GRP94 and FN14 pathways and GUILD, a network-based disease-gene prioritization program, to pinpoint the genes likely to be therapeutic targets, which resulted in FN14 as the main modulator and thalidomide as the best scored drug. The treatment of mice with brain metastasis improves survival decreasing reactive astrocytes and angiogenesis, and down-regulate FN14 and its ligand TWEAK. In conclusion our results indicate that FN14 and GRP94 are prediction/prognosis markers which open up new possibilities for preventing/treating brain metastasis.

## INTRODUCTION

Brain metastasis (BrM) occurs mainly after the diagnosis of systemic metastases in up to 30% of cancer patients [1, 2]. Despite the improvement in systemic therapies and the availability of more frequent imaging, central nervous system (CNS) relapse is emerging as an increasing clinical problem in up to 40% of cancer patients [3, 4]. The mean survival of these patients is 7 months [5, 6], posing CNS relapse a key research challenge.

The cross-subtype comparison involving both wet-lab and clinical studies reflects the heterogeneity of carcinomas to brain metastasis progression [7]. It is now recognized that breast cancer is composed of several subtypes [8–10]. The large numbers of differentially expressed genes in the five molecular subtypes of breast carcinoma confirm the diversity of the underlying biology [11]. These biomarkers include the estrogen receptor (ER), progesterone receptor (PR), and human epidermal growth factor receptor 2 (ErbB2 or HER2). ER and PR positivity define Luminal tumors A and B, whereas ErbB2 expression occurs in hormone positive and in negative tumors and basal tumors are characterized by the absence of these biomarkers. Moreover, the clear differences in metastatic potential between subtypes raise the question as to whether some tumors are “hardwired” to metastasize to the brain [12, 13]. Known predictive factors for BrM are: *(i)* overexpression of ErbB2, *(ii)* lack of hormone receptor expression, *(iii)* triple-negative subtype (TNBC) with ER and PR-negative and normal ErbB2, *(iv)* patient age under 50 years and *(v)* the presence of positive regional lymph nodes and lung metastases [14, 15]. The basal subtype has the worst prognosis (3–4 months) and ErbB2-negative/hormone receptor positive disease has the best prognosis (over 20 months). In a retrospective series of metastatic breast carcinoma (MBC) patients treated with trastuzumab, 52% of them succumbed to CNS progression although the non-CNS disease was stable or responsive [16].

Different primary tumor types exhibit remarkable differences in developing BrM. Both small cell and non-small cell lung carcinomas, kidney cancer and melanoma are the principal tumors with brain metastasis ability [3]. Alterations in the expression of several genes, including ST6GALNAC5, transforming growth factor-β, vascular endothelial growth factor, Serpine 1 and Timp 1 have been implicated in brain metastasis [17]. In lung cancer the genes mostly associated with brain metastasis are EGFR, KRAS mutation at codon 12 and several chromosomal imbalances [18].

Therefore, understanding the properties of brain-trophic tumor cells is essential to identify patients with risk of brain metastasis and to effectively prevent it [19]. A research priority is to delineate pathogenic mechanisms of metastasis to the brain that would enable the heterogeneity among tumors to differentiate between indolent and aggressive lesions.

Patients with ErbB2-positive or TNBC have an increased risk of BrM development [20, 21]. A recent study reported that both basal-like and claudin-low breast cancers exhibited a high probability of metastasizing to the brain and lung, while ErbB2-enriched tumors preferentially colonized the liver [12]. It has been observed that active WNT/b-catenin signaling contributes to the metastasis of basal breast tumors to the brain, whereas the absence of WNT/beta-catenin signaling allows luminal B-type tumors to metastasize to bone [13]. Moreover, a 13-gene signature predicting rapid development of brain metastases in patients with ErbB2-positive advanced breast cancer may be useful in the design of preventive therapies [19]. We identifiedan expression signature based on breast cancer BrM cells mapped onto an experimental protein–protein interaction network, which found 37 proteins differentially expressed in brain metastases [23]. The combination of GRP94, FN14 and TRAF2 expression, and the absence of Inhibin in breast carcinomas, referred to as the endoplasmic reticulum stress resistance phenotype (ERSRP), was the best signature for discriminating between breast carcinomas according to their BrM progression, regardless of whether or not they expressed ErbB-2 [24].

Bearing in mind that metastasis could already be underway at the time of diagnosis [25], ERSRP provides a predictive tool to help decide on treatment under the risk of BrM progression. We performed a multicenter study with breast carcinomas provided from three different hospitals and assessed ERSRP expression in tissue microarrays to independently validate it. Over-expression of GRP94 and FN14 in primary breast cancer was confirmed as a BrM predictor and the presence of FN14 and GRP94 in pairs of tumor/brain metastasis of lung and clear cell kidney carcinomas suggest that this phenotype might indicate BrM in tumors from different tissues. Moreover, we used bioinformatics tools such as BIANA [26] and GUILD [27] to prioritize genes implicated in BCBrM progression, and based on the network topology we assessed the level of association of genes with BCBrM (GUILD scores). We then ranked drugs using the GUILD score of their targets and produced a list of candidate drugs with high therapeutic potential, which were validated in preclinical experiments. Moreover, the preliminary experimental results suggested that the therapeutic activity of thalidomide derivatives might be dependent on brain organspecific microenvironmental factors produced by reactive astrocytes.

## RESULTS

### GRP94 and FN14 are biomarkers that predict brain metastasis progression in breast cancer patients

On the basis of retrospective observations, risk factors for the development of CNS metastases from breast cancer included patient characteristics and biological features of tumors such as ER negativity, ErbB2 positivity, a large primary tumor and loss of histopathological differentiation [35]. Among clinical parameters the presence of lymph-node metastasis is intrinsic to establishing the breast cancer prognosis [36]. We took all of these parameters into account when evaluating the contribution of the previously defined breast cancer brain metastasis biomarkers (BCBrMBK): GRP94, TRAF2, FN14 and Inhibin [24].

**Figure 1:**
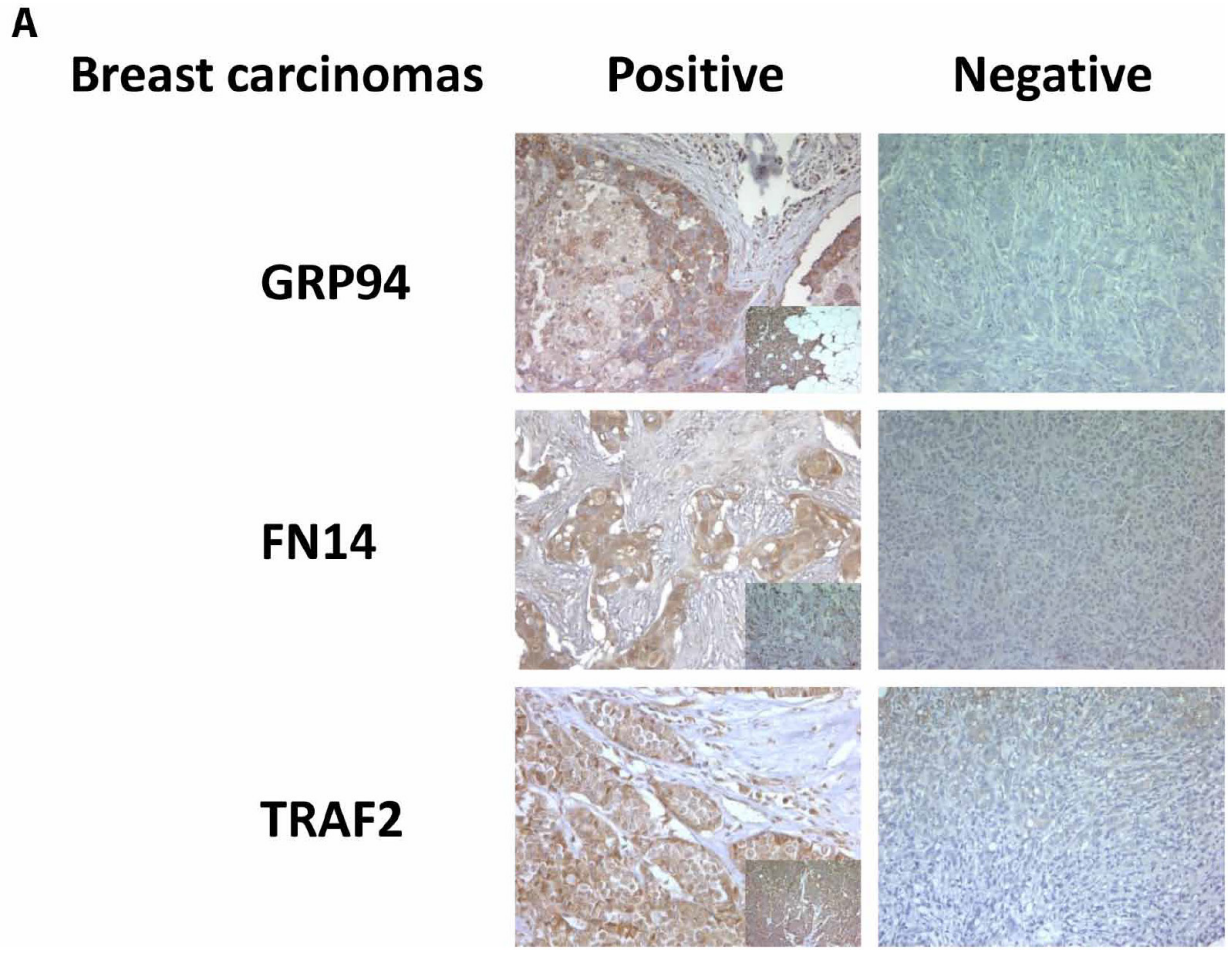

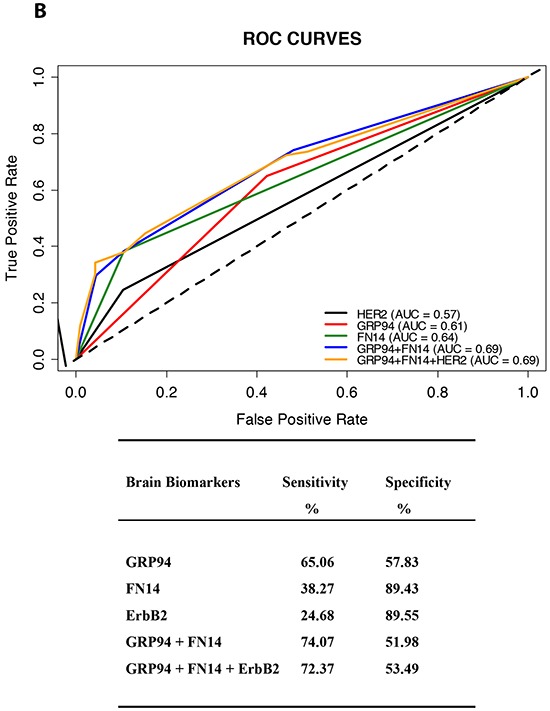

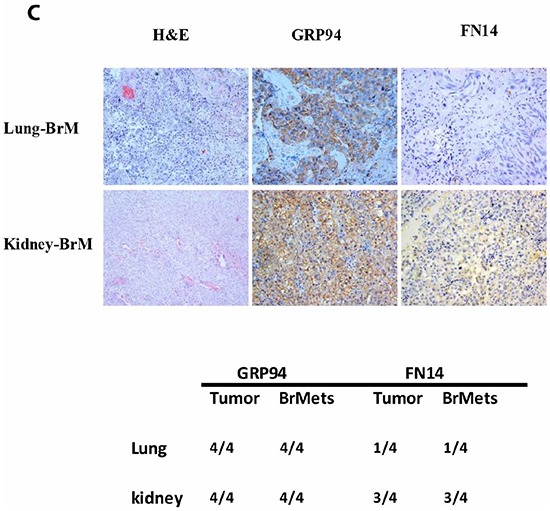
GRP94 and FN14 expression predict brain metastasis progression in breast cancer patients **A.** TMAs were used to identify the indicated proteins by IHC analysis in paraffin-embedded primary breast carcinomas (x 20). To score the positivity of the three proteins we considered samples with more than 70% of tumor positive cells with high levels of staining to represent positive control samples (small squares in the left column), ignoring samples with less intense staining or fewer positive cells. **B.** The area under the ROC curve obtained with the integrated predictive indexes. Markers were assessed in a multivariate logistic regression model using a forward stepwise procedure to identify the best combination for predicting brain metastasis. The area under the ROC curve obtained for ErbB2 alone (AUC = 0.57), for GRP94 (AUC = 0.61), FN14 (AUC = 0.64) and the combination of GRP94 and FN14 (AUC = 0.69) and for ErbB2, GRP94 and FN14 (AUC = 0.69), is represented in the upper part of the figure. The sensitivity and specificity of the markers are shown in the lower part of the figure, indicating the most specific GRP94 and the most sensitive FN14, which was similar to ErbB2 in terms of sensitivity and specificity. **C.** Expression of GRP94 and FN14 in four pairs tumor/BrM of lung and clear cell kidney carcinomas. Representative IHC of BrM are showed in the upper part of the figure and at the bottom the relation of positive samples of each protein.

The overexpression of BCBrMBK was categorized as positive when strong expression was detected in more than 70% of tumor cells (Figure 1A). On this basis, GRP94 was overexpressed in 48.2% (151/313), FN14 in 17.9% (55/308) and TRAF2 in 31.4% (97/309) of tumors. Inhibin expression, which was inversely associated with brain metastasis progression, was found in only 3.57% (11/308) of samples. The variation in the denominators is the result of taking into account the missing values in the immunohistochemistry data (tissue lost, unviable staining, background, etc.).

We first analyzed the association between the overexpression of proteins and the clinico-pathological features of the patients (Table 1). Patients with infiltrated axillary lymph nodes overexpressed GRP94 (Chi-square test, *p* = 0.049), whereas tumors with a high histological grade (III) were associated with FN14 expression (Chi-square test *p* = 0.004). The expression of both markers was associated with ErbB2 positivity (*p* = 0.013 and *p* = 0.005, respectively). Moreover, the expression of biomarkers was not associated with hormone receptors. The combination of negative hormone receptors and ErbB2 expression in TNBC patients was independent from the expression of GRP94 (*p* = 0.481) and FN14 (*p* = 0.914).

**Table 1:** Association between clinical and pathological characteristics and the expression of breast cancer brain metastasis biomarkers

	GRP94	FN14	TRAF2
	(*N* = 313)*	(*N* = 308)*	(*N* = 309)*
	No. Patients (%)**	No. Patients (%)**	No. Patients (%)**
	151 (48.2)	55 (17.9)	97 (31.4)
Tumor Size			
≤ 2 cm	78 (45.6)	24 (14.2)	55 (32.7)
> 2 cm	65 (52.8)	25 (20.7)	34 (27.7)
	*Chisq2 test p = 0.269*	*Chisq2 test p = 0.198*	*Chisq2 test p = 0.422*
Axillary Lymph Node			
0	78 (50.7)	23 (15.0)	44 (28.8)
1–3	30 (37.0)	13 (16.5)	25 (31.7)
≥4	37 (56.1)	15 (23.4)	22 (33.9)
	*Chisq2 test p = 0.049*	*Chisq2 test p = 0.320*	*Chisq2 test p = 0.738*
Histological Grade			
I	5 (31.3)	3 (18.8)	6 (37.5)
II	74 (51.8)	14 (9.8)	43 (30.5)
III	66 (50.0)	32 (25.2)	41 (31.5)
	*Chisq2 test p = 0.298*	*Chisq2 test p = 0.004*	*Chisq2 test p = 0.848*
Steroid Receptors			
Estrogen +	104 (46.9)	34 (15.5)	70 (31.8)
Estrogen −	37 (48.7)	18 (24.3)	23 (31.1)
	*Chisq2 test p = 0.886*	*Chisq2 test p = 0.124*	*Chisq2 test p = 0.979*
Progesterone +	87 (45.1)	33 (17.3)	57 (29.8)
Progesterone −	52 (51.5)	19 (19.4)	35 (35.4)
	*Chisq2 test p = 0.356*	*Chisq2 test p = 0.779*	*Chisq2 test p = 0.411*
ErbB2			
Positive	27 (65.9)	14 (35.0)	17 (42.5)
Negative	110 (43.7)	38 (15.1)	75 (29.9)
	*Chisq2 test p = 0.013*	*Chisq2 test p = 0.005*	*Chisq2 test p = 0.158*
Subtypes			
Triple Negative	18 (41.9)	7 (16.2)	12 (27.9)
Others	125 (49.0)	46 (18.3)	82 (32.5)
	*Chisq2 test p = 0.481*	*Chisq2 test p = 0.914*	*Chisq2 test p = 0.671*

* The variation in the denominators is the result of taking into account the missing values in the IHC data (tissue lost, unviable staining, background, etc.).

** The percentages of positive tumors distributed according to clinical and pathological characteristics of patients.

Statistical analysis of the data showed significant associations between BrM progression and high expression of GRP94 (*p* = 0.0004) and FN14 (*p* < 0.0001). The expression of TRAF2 was marginally associated with brain metastasis (*p* = 0.084). Inhibin was not correlated with BrM relapse (*p* = 0.428).

As expected, the ErbB2 expression detected in 14.4% (42/292) of patients (26 missing values) was significantly associated with BrM (*p* = 0.003): 24.7% (19/77) of breast cancers that progressed to brain metastasis were positive *versus* 12.8% (6/47) and 10.1% (17/168) of breast carcinomas that relapsed in other locations (such as lung, liver, bone or non-regional lymph nodes) or without metastasis, respectively. The incidence of BrM in patients with hormone receptors that expressed ErbB2 was 11.5%.

A total of 15.5% (46/297) of patients (21 missing values) were triple-negative (TNBC) with an increased risk of BrM progression (*p* < 0.0001). The incidence of BrM in this group was 58.7% (27/46); 15.2% (7/46) of patients had non-brain metastasis (lung, liver, bone or non-regional lymph nodes) and 26.1% (12/46) of patients did not progress to metastasis (*p* < 0.0001).

We found no correlation between lung, bone or liver metastasis and high levels of expression of BCBrMBK.

The overexpression of GRP94 in patients who developed brain metastasis was 65.1% (54/83), whereas GRP94 overexpression was found in 42.2% (97/230) in the other and non-metastasis group. FN14 was overexpressed in 38.3% (31/81) of brain metastasis relapse patients and only in 10.6% (24/227) of the others and non-metastasis group. The variation in the denominators is the result of taking into account the missing values of clinicopathological data.

The multivariate analysis comparing samples from patients who relapsed in the brain versus those who relapsed in other organs and without metastasis, indicated that the likelihood of relapse in patients with GRP94-positive tumors was 2.55-fold higher (95% CI 1.52–4.3, *p* = 0.0003) and increased to up to 5.24-fold higher (95% CI 2.83–9.71, *p* < 0.0001) if the tumors expressed FN14 (Table II). The combination of both markers was significantly associated to brain metastasis (*p* = 0.0014). These results indicated the predictive potential of both molecules for establishing the risk associated with a tumor that shows these characteristics. The overexpression of TRAF2 in tumors was associated with a 1.60-fold increased likelihood of relapse in the brain, although this increase was not significant (*p* = 0.086).

**Table 2:** Odds ratio for brain metastasis according to the expression of breast cancer brain metastasis biomarkers in primary tumors

	Metastases Incidence				
	BRAIN	OTHERS & NON MET	OR	(95% CI)	*p* - value
GRP94	54/83 (65.1%)	97/230 (42.2%)	2.55	(1.52 – 4.3)	*0.0003*
FN14	31/81 (38.3%)	24/227 (10.6%)	5.24	(2.83 – 9.71)	*< 0.0001*
TRAF2	32/82 (39.0%)	65/227 (28.6%)	1.60	(0.94 – 2.71)	*0.0859*

* The variation in the denominators is the result of taking into account the missing values in the IHC data (tissue lost, unviable staining, background, etc.).

When multivariate analysis was corrected by the covariables tumor size, histological grade, lymph nodes, hormone receptors, ErbB2, therapy (adjuvant chemotherapy and hormones), the best predictive markers for the presence of BrM were FN14 (*p* < 0.0001) followed by GRP94 (*p* = 0.0017). These results confirmed the independence of both biomarkers from the classical categorization of breast carcinomas and from the five breast cancer subtypes, and reinforce their intrinsic value as biomarkers for predicting BrM relapse, whereas TRAF2 and Inhibin were no longer significant (*p* = 0.481 and *p* = 0.736, respectively).

A multivariate analysis based on stepwise logistic regression retained GRP94 and FN14 as the best combination for predicting brain metastasis (Figure 1B). We calculated the positive and negative likelihood ratios to assess the predictive accuracy of each molecule as a BrM marker, considering the sensitivity and specificity of each. GRP94 was the most sensitive (0.65) and FN14 the most specific (0.89), and the combination of both increased the predictability of brain metastasis risk (AUC = 0.69) above that ascribed to ErbB2 overexpression (AUC = 0.57). Since FN14 had better sensitivity than ErbB2 (38.27% *vs*. 24.68%), the additional information on ErbB2 status did not improve the prediction of BrM in breast cancer patients (AUC = 0.69). Therefore, the expression of FN14 in primary tumors was by far the strongest predictor of the likelihood of BrM in breast cancer patients and could be used to stratify patients according to their risk of developing BrM, both for therapeutic decision making at first diagnosis and to indicate preventive treatments.

GRP94 and FN14 were also expressed in tumor metastasis brain pairs from non-small cell lung carcinoma and clear cell kidney carcinoma patients (Figure 1C). Both, FN14 and GRP94 expression in primary tumors and the corresponding brain metastasis, being GRP94 more sensitive to distinguish brain metastasis ability. In lung tumors FN14 expression might be only circumscribed to a subtype from those which develop brain metastasis.

### The expression of FN14 is a prognostic marker in breast cancer patients

**Figure 2:**
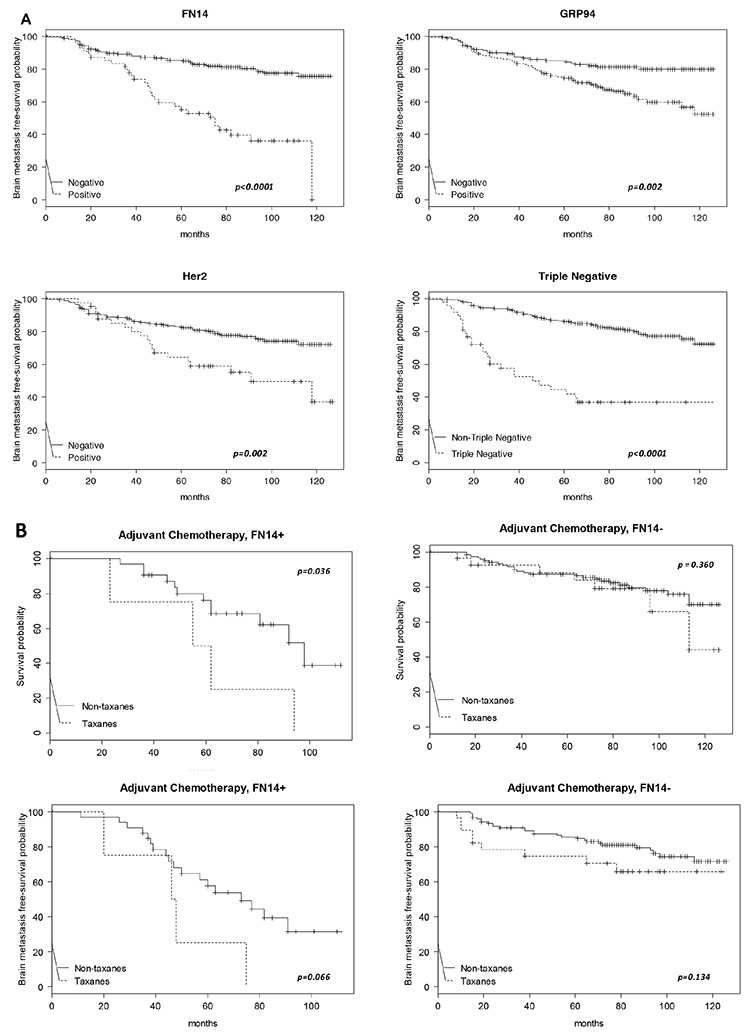
Kaplan-Meier survival estimates of brain metastasis-free survival among patients **A.** According to the expression of FN14 (total *N* = 308, events *N* = 81), GRP94 (total *N* = 313, events *N* = 83), ErbB2 (total *N* = 297, events *N* = 77) and TNBC (total *N* = 301, events *N* = 77). The *p* values were obtained from the log-rank test. **B.** Kaplan-Meier survival estimates of overall free survival (both upper panels) and brain metastasis-free survival (both bottom panels) among patients who received chemoadjuvant therapy, with or without taxanes, according to the FN14 expression in tumors.

The unpredictable clinical behavior of TNBC and ErbB2-positive tumors reflects the biological heterogeneity of the disease [19, 33, 37, 38]. We analyzed BrM-free survival (Figure 2A), the number of months from diagnosis of primary tumor to diagnosis of brain metastasis, and we correlated with patients according to whether the tumors were TNBC (bottom-right panel) or expressed ErbB2 (bottom-left panel), FN14 (upper-left panel) or GRP94 (upper-right panel). Patients with FN14-positive tumors (81/308, 10 deleted due to missingness), Figure 2A, had a shorter BrM-free survival than patients with negative tumors (*p* < 0.0001) and 31 developed brain metastasis; similar to that of TNBC patients (77/301, *p* < 0.0001). Patients with ErbB2-positive tumors (77/297, *p* = 0.002) and GRP94-positive tumors (83/313, *p* = 0.002) also had a significantly shorter BrM-free survival than patients whose tumors did not expressed the protein. These results confirmed that FN14 and GRP94 have prognostic value as widely accepted breast cancer prognostic markers.

Since adjuvant therapy is selected according to the biological features of the primary tumor, and as long-term efficacy implies the lack of disease relapse, we assessed whether FN14 and GRP94 predicted the response to adjuvant therapy by evaluating overall survival (Figure 2B). Interestingly, patients treated with taxanes survived a shorter period with regard to other therapeutic regimens (upper-left panel) when tumors expressed FN14 (16/37, *p* = 0.048). In contrast, the survival of patients with FN14-negative tumors (upper-right panel) was similar in those treated with taxanes or with other therapeutic regimens (36/150, *p* = 0.360). These results show that FN14 predicted taxane protocol failure in breast cancer patients, suggesting a relationship between FN14 expression and the shortening of BrM-free survival (*p* = 0.066) in patients treated with taxanes (bottom-left panel). In contrast, BrM-free survival was not associated with the therapeutic protocol in patients with FN14-negative tumors (bottom-right panel).

The association between FN14 expression and the efficacy of taxanes against breast cancer *in vivo* was explored by performing experiments with two TNBC breast cancer xenografts obtained from breast cancer patients that expressed (TNBC-EG) or did not express (TNBC-1070) FN14 (Supplementary Figure S1A, right panel and left panel, respectively). Although docetaxel treatment diminished the growth of tumors with regard to controls after 15 days of treatment in both models (Supplementary Figure S1B), the rate of increase of the tumor volume was reduced only 50% (*p* = 0.003) in FN14-positive tumors, whereas the rate of increase was reduced by 85% (*p* < 0.001) in FN14–negative tumors with regard to the control.

The survival of patients given adjuvant treatments, either taxanes or other chemotherapeutic protocols (Supplementary Figure S2), was not correlated with GRP94 expression in tumors, whether positive (*N* = 98) or negative (*N* = 92), *p* = 0.31 and *p* = 0.29, respectively. These results indicate GRP94 and FN14 are involved in different pathways and functions in BrM progression, suggesting that both biomarkers might be therapeutically inhibited.

### Modeling personalized therapies to prevent brain metastases using GRP94 and FN14 pathways

The remarkable diversity in breast cancer dictates that adjuvant management must be biologically driven [39], and thus early breast cancer assessment with tools for prognosis and prediction of treatment benefit may aid clinical decision making. Indeed, an important question is how to identify the specific adjuvant interventions that would improve the prognosis of BC patients with risk of BrM progression. Since the pathophenotype is the outcome of perturbations in the underlying regulatory pathways, we designed experiments to highlight the usefulness of BCBrMBK expression in choosing the most appropriate adjuvant therapy.

We have previously cataloged the organ-specific metastasis signature (BOSMS) with a hierarchical clustering containing 1,193 genes after one-versus-all (ONA) class comparisons, which clearly distinguished between the different metastases [40]. These datasets, under the identification number GSE11078, are freely available from the Gene Expression Omnibus (GEO) repository. The BOSMS mapped human brain metastasis expression profiles with a PPIN to maximize accuracy in the classification of brain metastasis proteins and permitted the identification of protein folding and chaperones connecting different functions and performing the endoplasmic reticulum stress resistance phenotype (ERSRP) [24]. Indeed, rather than having random connections through the network, the interaction of proteins encoded by genes implicated in such phenotypes involves partners from similar diseases [26]. We used these data, together with systems biology and computational approaches, to create treatment strategies using the BCBrMBK. First we listed the brain organ-specific genes/proteins [24, 41], including up- and downstream molecules of signaling pathways connected functionally with GRP94 and FN14 (Supplementary Table S1). Using GUILD, a bioinformatics software [28], the brain organ-specific genes and the PPIN, we prioritized the genes for BCBrMK based on the network topology. The best-positioned molecule in the ranking was FN14, followed by TRAF2, TANK, TP53 and HSP90B1 (GRP94) in fifth position (Supplementary Table S2).

**Figure 3:**
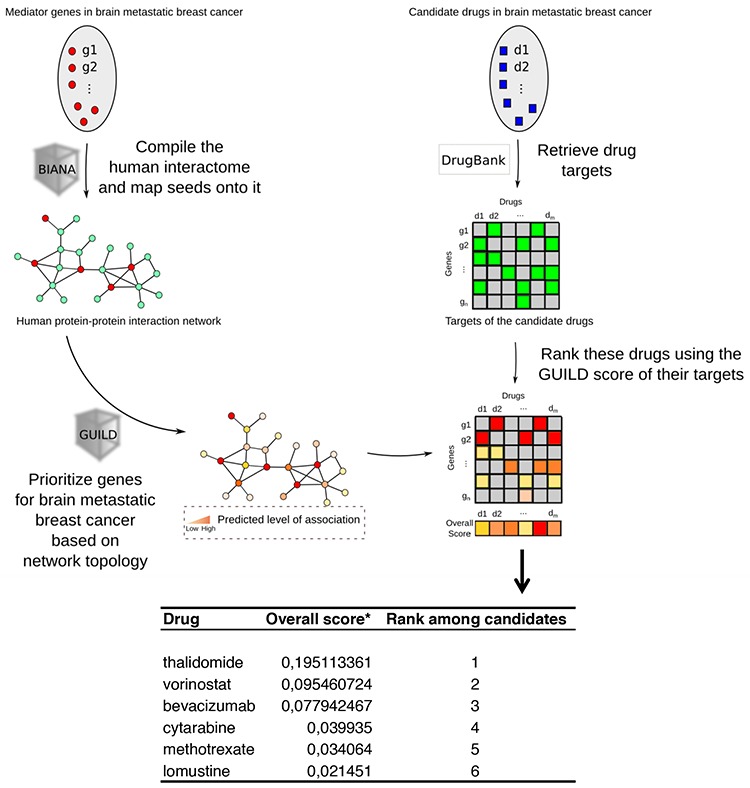
Modeling personalized therapies to prevent brain metastases using GRP94 and FN14 pathways as targets Workflow chart describing the process of prioritizing genes involved in breast cancer brain metastasis based on network topology, retrieval of drugs from the DrugBank and ranking of these drugs using the GUILD score to determine the best treatment. The list of targets prioritized is shown at the bottom, indicating that thalidomide is the best drug to target FN14.

Candidate drugs in brain metastatic breast cancer were retrieved from the DrugBank and drugs were ranked using the score for brain metastasis proteins (Figure 3). As shown at the bottom of Figure 3, the most specific drugs intercepting the BCBrMBK pathways are thalidomide, overall score (*o.s*.) = 0.195, vorinostat *o.s*. = 0.095 and bevacizumab *o.s*. = 0.078.

The ranking of molecules under thalidomide therapeutic action included TNFα as the best therapeutic target in position 194. Other proteins linked to thalidomide therapy were positioned downstream: PTGS2 (246), NFKB1 (1326), and FGFR2 (5800), (Supplementary Table S3).

Other drugs retrieved from the DrugBank for the drugs in the Medtrack file were: cytarabine, lomustine and methotrexate, which mechanism of action involved POLB, STMN4 and DHFR, respectively (Supplementary Table S3). Temozolamide was retrieved without a known molecule. Heat shock inhibitors were retrieved from the DrugBank below position 8659, the rank of bevacizumab action over the VEGFA protein.

### Targeting FN14 with thalidomide derivatives improves brain metastasis outcome in preclinical experimental models

Since GUILD retrieved FN14 as the main regulator of BrM protein-protein interacting network, we hypothesized that thalidomide therapy could impair FN14/TWEAK function in the BrM.

It is well known that FN14 belongs to the TNFR family and is activated by its specific ligand TWEAK and by TNFα [42]. Among other cytokines TWEAK is expressed in brain, mainly produced by astrocytes and microglial cells [43]. The binding of TWEAK to FN14 has a direct effect on the composition of the basal lamina (BL) and on the perivascular astrocytes (PA), regulating their interaction with endothelial cells (EC) and regulating the function of the BBB in the EC-BM-astrocyte interface [44].

**Figure 4:**
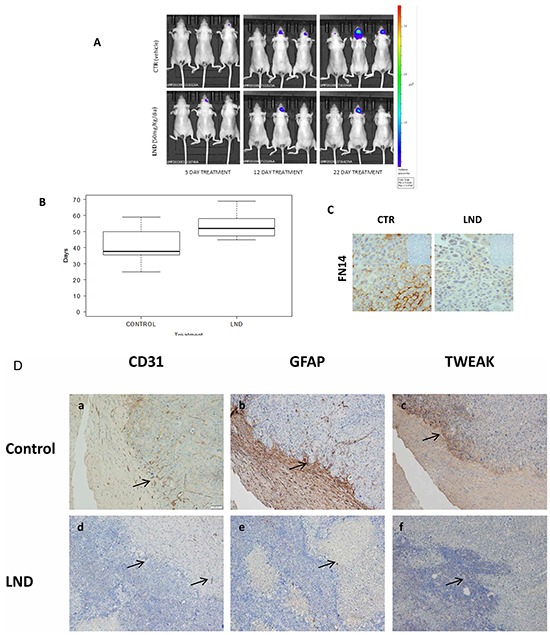

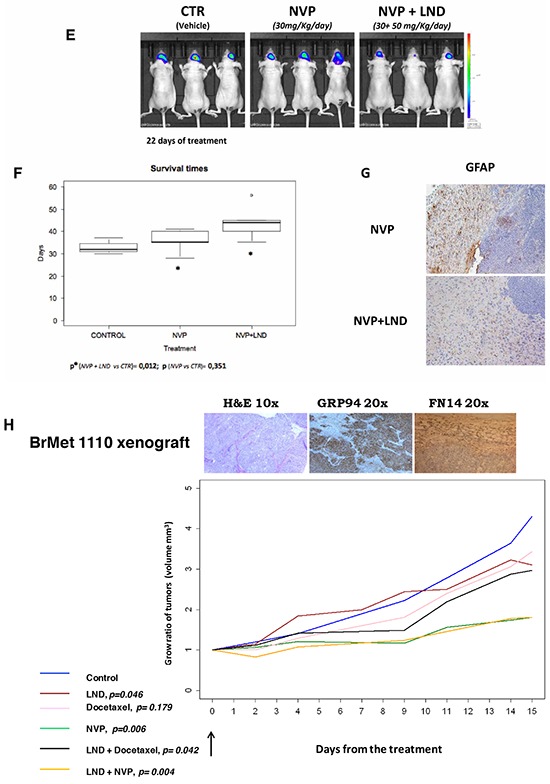
Experimental brain metastasis models to validate the therapeutic effect of a thalidomide derivative Lenalidomide, 50 mg/Kg/day, alone or in combination with Docetaxel 15 mg/Kg/day and NVP-AUY922 (NVP) 30 mg/Kg/day. Docetaxel was administered every 4 days for 2 weeks and NVP-AUY922 every 2 days for 2 weeks. **A.** Representative images show bioluminescence in animals at day 5, 12 and 22 from the start of LND treatment 50 mg/Kg/day every day (Celgene Corporation, Summit, NJ). **B.** The survival time (box plot) of mice treated with LND (*N* = 8) or with vehicle (Control, *N* = 8) was evaluated using the log-rank test (*p* = 0.0802) and the differences observed in the survival rate between the two groups (treated or not with LND) are represented. **C.** IHC analysis in paraffin-embedded experimental brain metastasis (x 20) shows the downregulation of FN14 in mice treated with LND. **D.** IHC analysis in paraffin-embedded experimental brain metastasis (x 20). Representative figures showing the upfront of the metastasis invading the brain tissue in controls (upper part of the figure). Each protein expressed is indicated. CD31 **a.** GFAP **b.** and TWEAK **c.** Decreased angiogenesis **d.** few reactive astrocytes **e.** and downregulation of TWEAK f. indicated with arrows in tissues from mice treated with LND with regard to controls (lower part of the figure). **E.** Representative images show bioluminescence in animals at day 22 from the start of indicated treatments. The bioluminescence data were transformed using the log(1+x) function (where x = AvR), in order to obtain a more regular and positive distribution. Subsequently, these data were normalized by subtracting the first observation (day 14) from each of the following observations. **F.** The survival time (box plot) of mice treated with NVP-AUY922 (*N* = 6) alone or in combination with LND (*N* = 6) are shown. The differences in mouse survival were statistically significant in NVP-AUY922 + LND vs. CTR (*p* = 0.012) and NVP-AUY922 alone vs NVP-AUY922 + LND (*p* = 0.033); but the survival of mice treated only with NVP-AUY922 and controls was similar (*p* = 0.351). **G.** IHC of brain metastasis from mice treated with NVP-AUY922 alone and in convination with LND. The expression of GFAP indicative of reactive astrocytes is higher in NVP-AUY922 + LDN group than in brain metastasis from mice treated with NVP-AUY922 alone. **H.** Athymic Nude-Foxn1nu female mice. The xenograft expression of GRP94 and FN14 is shown in the upper part, in tumoral cells (GRP94) and in tumor and estromal cells (FN14). The statistical differences between groups of treatment (*): control treated with vehicle (N=17), Docetaxel (N=10), LND (N=16), NVP (N=15), NVP+LND (N=9) and Docetaxel+LND (N=20) according the volume slope are indicated at the bottom of the figure.

To analyze if LND can impair FN14/TWEAK *in vivo*, we performed experiments to inhibit BrM growth in a classical model of brain metastasis [30], where the proprietary cells BRV5eGFP-CMV/Luc (BrV5), obtained from 435Br1 cells by *in vivo*/*in vitro* selection, were injected intra-cranially by stereotaxia to homogenize the groups of treatment. The cellular burden was followed by luminescence to assess the volume slope of intracranial masses in animals treated with the vehicle or with a thalidomide derivative lenalidomide (LND), the weight of the animals was monitored three times a week (m-w-f) and the bioluminescence analysis was done twice a week (m-f) until the symptoms of metastasis appeared. The differences observed in the increasing rate of bioluminescence between the two groups (treated or not with LND) were not statistically significant (Student test, *p* = 0,39), due in part to the heterogenic growth of cells (Figure 4A). In addition, we analyzed mice survival and the results showed moderate differences (Figure 4B) between treated and non-treated mice (*p* = 0.080). The IHC analysis of brain tissues showed that LND decreased the expression of FN14 in tumoral cells (Figure 4C).

These results fit with the survival of 435Br1 cells stimulated with TWEAK (Supplementary Figure S3A and S3B), which increased with regard to parental cells, suggesting the high functionality of the FN14/TWEAK pathway. Moreover, we found an IC_50_ > 100 μM when challenged 435Br1 cells with LND. Therefore, LND does not exert *in vitro* cytotoxicity against carcinoma cells, as was the case in human myeloma cell lines challenged *in vitro* with the cell-extrinsic component of the antineoplastic activity of LND, which prevails over its cell-intrinsic counterpart [45]. In contrast, 435Br1 cells overexpressed GRP94 (Supplementary Figure S4A), and the heat shock protein inhibitor (NVP-AUY922) [46] was highly cytotoxic and the IC_50_ was 10–20 nM (Supplementary Figure S4B).

We analyzed the contribution of the CNS microenvironment in brain metastasis from treated and control mice. The expression of reactive astrocytes around cancer cells diminished together with the down regulation of TWEAK and CD31 expression in treated mice with regard to controls (Figure 4D). These results indicated that LND had a microenvironmental action in BrM that can involve astrocyte response and angiogenesis.

Furthermore, we analyze LND activity in adjuvancy with the cytotoxic drug, synchronizing the same scheme of LND treatment with NVP-AUY922 (Figure 4E and 4F). The mice had a significantly lower tumor burden slope than those treated with the vehicle or with NVP-AUY922 alone and the survival of mice differed significantly between groups (NVP-AUY922 + LND *vs*. CTR, *p* = 0.012; NVP-AUY922 + LND *vs*. NVP-AUY922, *p* = 0.033). In contrast, the survival of mice treated with NVP-AUY922 alone and control mice was similar (NVP-AUY922 *vs*. CTR, *p* = 0.351). Reactive astrocytes decreased according to the expression of GFAP in NVP-AUY922 + LND with regard to NVP alone (Figure 4G). These results suggested that the therapeutic effect of LND might contribute to the success of adjuvant treatment by decreasing reactive astrocytes and/or modifying the BBB penetration [44].

In addition, to avoid BBB permeability maintaining the stromal and phenotypic characteristic of brain metastasis tissue, we treated a group of mice with a subcutaneously engrafted brain metastasis biopsy, which expressed both GRP94 and FN14 proteins, obtained from a woman with primary lung carcinoma that metastasized in the brain. We compared tumor growth in mice treated with LND, docetaxel and NVP-AUY922 alone or in combination with LND (Figure 4H). LND alone (*p* = 0.046) reduced the engrafted brain metastasis in mice, even more in combination with docetaxel (*p* = 0.004), in contrast to docetaxel alone (*p* = 0.179). These preliminary results showed that LND might improve therapy responses in brain metastatic tissue. Moreover, NVP-AUY922 decreased graft development alone (*p* = 0.006) and in combination with LND (*p* = 0.004). In this case the high cytotoxicity of the drug is manifested when BBB is avoided.

These preliminary results suggested that the action of thalidomide derivatives could impair FN14/TWEAK axis through its reactive astrocytes activity. In fact, LND did not reduce the tumor slope of i.m.f.p. breast cancer tumors induced with EG and 1070 TNBC breast carcinomas (Supplementary Figure S5), even thought decreased the expression of FN14 positive EG tumors, probably intercepting other stromal cytokines production.

## DISCUSSION

To our knowledge, this study is the first to report that fibroblast growth factor-inducible protein 14 (FN14) expression and GRP94 expression in a patient diagnosed with breast cancer (BC) indicates a high risk of brain metastasis (BrM) progression, offering the opportunity to develop therapeutic strategies either to prevent the disease or facilitate early detection. Moreover, other tumors like lung carcinomas and clear cell kidney carcinomas might express these biomarkers according their ability to develop BrM.

The major challenge for both primary and secondary prevention studies is the identification of patients at highest risk of developing brain metastasis due to tumor and host factors. For cancers of the breast, BrM occurs after diagnosis of systemic metastases from tumors belonging to one of two categories: tumors with amplification of ErbB2 or TNBC; the incidence of both exceeds one-third of patients [47, 48]. We demonstrate that FN14 and GRP94 are independent prognostic factors and give information on the likelihood that patients will develop brain metastasis, either in ErbB2 or TNBC patients.

FN14 positivity was associated with ErbB2 expression in our series, consistent with reports that high FN14 expression levels were significantly correlated with several poor prognostic indicators, being higher particularly in ErbB2-positive breast cancer [49]. Moreover, FN14 positive tumors might have a poor survival because they are likely to develop brain metastasis. On the other hand, GRP94 correlates with ErbB2 expression and poor BrM free survival of breast cancer patients. Indeed, GRP94 specific inhibitors provided evidence for the role of GRP94 in maintaining the architecture of high-density ErbB2 formations at the plasma membrane, which is vital for proper ErbB2 functioning [50].

FN14 is a small cell-surface protein that might modulate cell–extracellular matrix interactions, the expression of which is frequently found to be strongly enhanced in tumor tissue compared to non-transformed tissue [49]. The tumor microenvironment typically contains many factors implicated in the upregulation of FN14 expression [51]. High FN14 has been found in tissues damaged by different insults including hypoxia, oxidative stress, chemical and mechanical injuries and tumor growth [52]. Although the regulatory elements involved in FN14 gene activation have not yet been elucidated, the human FN14 promoter region contains several potential transcription factor binding sites, including AP-1 sites and the NFkB site [53]. RNA interference-mediated inhibition of FN14 expression in metastatic MDA-MB-231 breast cancer cells reduces invasion through activation of the NF-kB pathway [49]. Moreover, FN14 has been shown to promote breast cancer cell migration, invasion and MMP9 expression [54]. These data illustrate the importance of FN14 expression in mechanistic pathways of metastasis progression.

Brain colonization involves many factors implicated in the upregulation of FN14 expression. Among them, reactive astrocytes [55] represent a bona fide source of TWEAK (TNF-like weak inducer of apoptosis), its only known signaling-competent receptor [51]. Further work is required to unraveling the important role that FN14/TWEAK axis in different pathogenic steps of brain metastasis progression, including BBB permeability.

Consistent with the hypothesis that LND action might involve the physical and tropic interaction between cancer cells and the CNS environment, the treatment of experimental brain metastasis resulted in a reduction in tumor volume with reactive astrocytes and angiogenesi decrease and down-regulation of FN14 and TWEAK expression. A similar therapeutic effect has been reported with the use of immunotoxins targeting the FN14 receptor [56]. Indeed, a phase I study of RG7212, a humanized anti-TWEAK IgG1k monoclonal antibody, was conducted in patients with advanced solid tumors expressing FN14 resulting in tumor regression and prolonged stable disease [57]. This is the first evidence of a specific treatment against FN14/TWEAK that encourage the use of these molecules as a therapeutic target to develop new drugs to treat/prevent brain metastasis.

LND is a synthetic derivative of thalidomide currently approved by the US Food and Drug Administration for use in patients affected by multiple myeloma (in combination with dexamethasone) and low or intermediate-1 risk myelodysplastic syndromes that harbor 5q cytogenetic abnormalities [58]. Therefore, our results illustrate a new indication of LND for the treatment of brain metastasis when the primary tumor expresses FN14. Pomalidomide (CC4047) is a new thalidomide derivative with high *in vitro* potency. A first phase 1b, single-center, ascending dose study was conducted to identify the maximum tolerated dose (MTD) and evaluate the safety and efficacy of CC-4047 in relapsed or refractory LND-treated MM patients (http://ClinicalTrials.gov, number NCT01311687, and with EudraCT, number 2010–019820-30), showing a significantly longer median overall survival time with refractory or relapsed multiple myeloma patients [59].

BrM are remarkably heterogeneous in permeability to the chemotherapeutic agents, typically undergo higher levels of drug penetration than surrounding normal tissue, but far lower levels of penetration than metastases to other organs [60], due of drug efflux pumps for chemotherapeutic and molecular therapeutic agents [17]. Since St Gallen Consensus [61] is indicating cytotoxic drugs in TNBC, due to the limit distribution of taxanes to subtherapeutic levels in brain, it could be beneficious to treat FN14 positive tumors with drugs that have better penetration to CNS. This consideration might prevent to render the brain a “sanctuary site” for metastatic cells in TNBC patients.

On the other hand, in all meta-analyses involving taxane-based regimens or anthracycline-based regimens [62], the proportional reductions in early recurrence, any recurrence, and breast cancer mortality appeared largely independent of age, nodal status, tumor diameter, tumor differentiation (poorly or moderately differentiated, relatively few were well differentiated) or ER status (ER-poor or ER-positive). Indeed, for a patient with early-stage breast cancer, recommendations regarding systemic therapy and the most appropriate choice of agent(s) are often difficult (Early Breast Cancer Trialists' Collaborative Group (EBCTCG)). In this scenario, FN14 over-expression is an early event, which reflects specific mechanisms of breast cancer progression, correlates with clinico-pathological features and predicts BrM outcome in patients treated with protocols including taxanes. Therefore, FN14 combines prediction and prognostic information to stratify patients at first diagnosis according to the likelihood of BrM with accuracy similar to ErbB2, and thus it might help clinicians in deciding the therapeutic protocols to be adopted.

To date only ErbB2-positive breast cancer patients have entered prevention trials to clarify the role of lapatinib [63]. Most patients with BrM are suffering from terminal cancer, and control of brain metastasis is crucial for their quality of life. ErbB2-positive BC has seen more therapeutic progress than TNBC. The LANDSCAPE trial is a phase II study testing lapatinib plus capecitabine in previously untreated BCBrM and was positive for its primary endpoint, with 65.9% of patients presenting a partial response. In the light of these results we suggest that patients with triple-negative tumors and pulmonary metastasis might be the most suitable group for prospective trials investigating strategies for BrM screening and prevention. Indeed, FN14 and GRP94 might be a companion diagnostic markers that could identify patients who are likely to respond to drugs interfering with these specific targets.

Progress in treating brain metastases has been hampered by a lack of model systems, a lack of human tissue samples, and the exclusion of brain metastatic patients from many clinical trials. The Response Assessment in NeuroOncology (RANO) group has recently published the endpoints and trial consideration in brain metastases clinical trials [64]. This is clearly but the beginning of such considerations.

BrM therapy faces the challenge of efficiently targeting cancer cells or/and their supportive relationship with the brain parenchyma. Nowadays, the treatment armamentarium consists of a multimodality approach, selected according to the patient's symptoms and extent of disease. A systemic therapy might even prevent brain colonization altogether or at least arrest single cells or micrometastases in the dormant state. The role of thalidomide derivatives in preventing BrM progression is an ongoing investigation in our laboratory, with a particular focus on their hypothetical mechanism of action which might be associated with the regulation of immunoreactive astrocytes in the area surrounding BrM cells.

## MATERIALS AND METHODS

### Sample collection

We recruited samples from 318 patients diagnosed between 1989–2009 in three hospitals: 252 samples from the Catalan Institute of Oncology (I.C.O.) – Hospital Duran i Reynals and the Hospital Universitari de Bellvitge (L'Hospitalet de Llobregat, Spain); 24 samples from the Consorci Hospitalari Parc Taulí (Sabadell, Spain) and 42 samples from I.C.O. – Hospital Universitari Germans Trias i Pujol (Badalona, Spain). The patients were between 24–88 years old at diagnosis and 61% were diagnosed when in/older than their fifties (mean: 55 years). Follow-up ranged from 8 to 146 months (mean: 76.6 months). Metastasis relapse occurred in 43.4% (138/318) of patients; of these, 84 patients (60.9%) developed BrM, 47 (34.1%) lung metastasis, 54 (39.1%) liver metastasis, 40 (29.0%) non-regional lymph node metastasis and 89 (64.5%) bone metastasis. Just over half (56.6%; 180/318) of the patients had no metastatic progression after a minimum follow-up of 5 years.

In terms of histological type, the ductal type was identified in 94.1% (*n* = 299) of patients, followed by lobular in 5% (*n* = 16), mucinous in 0.6% (*n* = 2) and medullary in 0.3% (*n* = 1). Estrogen receptors (ER) were analyzed in 304 patients, being positive in 74.7% (*n* = 227); relevant data were missing in 14 patients. Progesterone receptors (PR) were evaluated in 299 patients (missing in 19), being positive in 65.9% (*n* = 197). ErbB2 scoring was obtained for 296 patients (22 cases missing), only 14.2% (*n* = 42) of whom were positive. The triple-negative status was analyzed in 302 patients (16 cases missing), with only 15.6% of them (*n* = 47) being identified as triple-negative.

Sixty-three percent (193/307) received adjuvant chemotherapy: schedules with CMF (*n* = 68), anthracyclines (*n* = 32), anthracyclines plus CMF (*n* = 60), taxanes (*n* = 21) and anthracyclines plus taxanes (*n* = 12); 6 patients were missing and 5 were not undergone to surgery. Only 3 patients received trastuzumab as adjuvant therapy. Twenty-nine percent (91/314) of patients received neoadjuvant chemotherapy and adjuvant hormonotherapy was prescribed in 65.1% (203/312); 1 case was missing and 5 not undergone to surgery. The variation in the denominators is the result of taking into account the missing values in the clinical-pathological parameters.

### Tissue microarrays (TMAs) and immunohistochemistry (IHC)

TMAs were prepared from three representative areas of the tumor that were carefully selected from hematoxylin-eosin-stained sections of the donor blocks. Core cylinders of 1 mm diameter were punched from each tumor using a skin-biopsy punch and deposited into recipient paraffin blocks using a specific arraying device (Beecher Instruments, Sun Prairie, WI) as described elsewhere [24]. Three-μm sections of the resulting tissue microarray block were cut and used for IHC analysis after being transferred to glass slides.

To optimize each immunohistochemical analysis, the corresponding control tissues for the expression of each protein were also used. Antigens were retrieved by heating in a pressure cooker for 7 minutes in the appropriate buffer. Primary antibodies anti-GRP94 at 1/2000 (sc-1794) and anti-FN14 at 1/3000 (sc-27143), both Santa Cruz Biotechnology (Santa Cruz, CA) and anti-TRAF2 at 1/100 (SM7106P, Acris Antibodies, Herford, Germany) were diluted in Dako Real™ Antibody Diluent Buffer (Dakocytomation; Dako, Glostrup, Denmark): Tris buffer, pH 7.2, 15 mM Na_3_N. LSAB+System-HRP (Dakocytomation) was used, including biotinylated anti-rabbit, anti-mouse and anti-goat immunoglobulins in PBS; streptavidin conjugated to HRP in PBS; and liquid 3–3′ diaminobenzidine in chromogen solution. The polyclonal antibody anti-ErbB2, A0485 (Dako) was used with the Ultraview detection kit in an automatic staining system (Benchmark XT, USA).

Staining optimization, evaluation parameters and analyses were established by two pathologists who were blinded to the clinical status. The overexpression of GRP94, FN14 and TRAF2 was categorized as positive when strong expression was detected and negative when no or weak expression was detected, in order to avoid false positives (Figure 1A), taking into account the known expression in a control tissue, as previously reported [24].

Four tumor-metastasis pairs of kidney and lung carcinoma from paraffin archives were used to explore the expression of biomarkers in tumors different from breast.

### Prioritization of brain metastasis candidates using protein–protein interactions

First, a protein–protein interaction subnetwork (PPIN) of the whole human interactome was built around a set of proteins known to be crucial for brain metastatic growth (i.e., root proteins). All the interactions of root proteins were retrieved from BIANA, provided that the interaction had been identified using an experimental method other than a pull-down method. Though suitable for defining protein complexes, interactions from pull-down methods might introduce spurious binary interactions between proteins. Then, gene expression levels were mapped onto the network. A protein was considered to be differentially expressed if the gene encoding for it was differentially expressed in the tissue microarray experiment. This mapping allowed us to find the active subnetworks: clusters of the network with a significant proportion of proteins produced by up- and down-regulated genes. Next, we calculated a brain metastasis likelihood score for all the nodes in the human interactome using GUILD [27], a network-based disease-gene prioritization software. GUILD assigns a disease-implication score to each node in the network by disseminating information about roots (known metastatic growth proteins) to other nodes through the links in the network. It can also be used to prioritize drugs for their potential to intervene in a given disease by considering the scores of the drug targets [28]. We used 15 root proteins (selected from those identified by proteomics analysis) for which we found interactions in the human interactome (see Supplementary Table S1). We applied the NetCombo algorithm implemented in GUILD, which produces a consensus score that considers the distance from the nodes to the roots in the network.

Next, we used the GUILD scores assigned to proteins to rank candidate drugs (listed in Supplementary Table S4). For each drug, we calculated the average GUILD score of its known targets. Drug-protein information was retrieved from the DrugBank database [29].

### Animal models

Athymic Nude-Foxn1nu female mice weighing 22–28 g were purchased from Harlan Laboratories S.A. (Barcelona, Spain) and were housed in the IDIBELL facility in SFP conditions, at 20–24°C, 60% relative humidity, and 12–12-hour light-dark periods. Animals were allowed free access to UV-irradiated water and an adequate sterile diet. All animal-related procedures were performed in accordance with the National Institute of Health Guidelines for the Care and Use of Laboratory Animals, with the approval of the animal care committee.

### Xenografts from primary tumors and brain metastasis biopsies

Samples were collected at Hospital Universitari de Bellvitge (L'Hospitalet de Llobregat, Barcelona, Spain). The study was approved by the Institutional Review Board. Written informed consent was collected from patients. Non-necrotic tissue pieces (2–3 mm^3^) from resected ductal breast carcinoma (TNBC-EG and TNBC-1070) or brain metastasis biopsies (BrM1110) were placed in DMEM (BioWhittaker) supplemented with 10% FBS and penicillin/streptomycin. The xenografts were implanted in animals under isofluorane-induced anesthesia, either in the intramammary fat path (i.m.f.p) or subcutaneously when the biopsy belonged to primary breast carcinoma or brain metastasis, respectively. When the i.m.f.p. tumors reached ~1000 mm^3^, they were excised, dissected into 2–3 mm^3^ cubes and transplanted into additional mice using the same procedure.

### Brain metastasis *in vivo* experiments

To induce brain metastases the highly brain metastatic cell line BR-eGFP-CMV/Luc-V5CA (BRV5) [30] was intracranially implanted [31]. In brief, 435-Br1 cells originally established from a brain metastasis in a *nude* mouse orthotopically inoculated with the triple negative MDA-MB 435 parental cell line [32], contain the retroviral vector preGFP-CMV-PLuc with the enhanced green fluorescent protein (*eGFP*) gene, under the control of the 5′ LTR, and the photinus luciferase (*PLuc*) gene, under the control of the cytomegalovirus (CMV) promoter [33]. Vector preparation and packaging of viral particles was performed as described previously. A cell population that uniformly expressed the highest levels of eGFP (BR-eGFP-CMV/Luc) was selected by FACS (MoFlo, Cytomation, Dako, Denmark). Left ventricle (*LV*) injection of cells and their further isolation from mouse brain was repeated five times, obtaining BR-eGFP-CMV/Luc-V1 to V5 cells through these cycles.

The controversial use of MDA-MB 435 cells has been recently clarified, since it has been demonstrate that MDA-MB 435 cells are an useful breast cancer model that expresses both, epithelial and melanocytic markers [34].

Animals were anesthetized by intraperitoneal injection of ketamine/medetomidine, with a previous subcutaneous injection of buprenorphine, and placed in a stereotactic apparatus. An incision was made over the cranial midline with a scalpel, and a hole 1 mm posterior to the bregma and 2 mm to the right of the midline was made with a 25 gauge needle by hand. Two microliters of the cell suspension at 10^7^ cells/ml in HBSS was infused with a Hamilton^®^ 10 μl syringe (Bonaduz, Switzerland) and a Hamilton^®^ needle (ga26s/51mm/pst2). Finally, the incision was sutured and medetomidine was added as the antidote. Animals were imaged and weighed three times within a week (on Monday, Wednesday and Friday), and were euthanized when they showed signs of declining health and visible body weight loss.

### *In vivo* bioluminescence imaging

Mice were injected subcutaneously with 10 μl/g body weight of D-Luciferin (Biosynth AG) 15 minutes before imaging. We anesthetized the animals with 4% Isofluorane gas in 2 l/min O_2_ and maintained anesthesia inside the chamber with 2% Isofluorane at 2 l/min O_2_ during acquisition. The animals were placed in the prone position. Cells showing bioluminescence were detected and quantified using the Living Image 4.1 image analysis software (Caliper, LifeSciences Hopkinton, MA). The parameter chosen for treatment evaluation was the total flux (p/s).

The background signal of each mouse (photons emitted by the mouse before the injection of luciferin) was subtracted in every bioluminescence cranial measurement.

The bioluminescence analysis was conducted once a week during the early stages of the disease and twice a week when brain masses began to grow exponentially.

### Histology and immunohistochemical tumor characterization in mice

The morphology of the engrafted tumors and brain metastases was analyzed by H&E staining in paraffin-embedded sections. Determination of GRP94, FN14 and TRAF2 was performed as described previously. IHC analysis with anti-CD31 (Dako) at (1/100), anti-TWEAK (Abcam, Cambridge, UK) at (1/300) and anti-GFAP (Dako) at (1/16000) were performed.

### Therapeutic protocols

For brain metastasis treatment, we started therapy on day 14 once the mice had recovered from surgery and after checking the success of cell inoculation.

Lenalidomide (LND), a thalidomide derivative, was obtained from the Celgene Corporation (Summit, NJ) and from Sellek Chemicals LLC (Houston, TX). LND was injected intraperitoneally in DMSO (Sigma-Aldrich) at 50 mg/Kg/day, every day until the end of the experiment. Docetaxel (TXT) and NVP-AUY922 (NVP), both from LC Laboratories, were injected intraperitoneally in DMSO at a dosage of 15 mg/Kg/day and 30 mg/Kg/day respectively. TXT was administered every 4 days for 2 weeks and NVP every 2 days for 2 weeks.

### Statistical analysis

To evaluate the correlation between protein expression and brain metastasis in patients, immunostained samples were graded on a three-category scale (negative, weak positive, and strong positive). The marker was classed as being overexpressed in strong positive samples. The association with brain metastasis for each marker was tested using a two-sided Fisher exact test and summarized by calculating the sensitivity among tumors that developed metastasis, and specificity among tumors without metastasis, for strong positive values. Positive and negative likelihood ratios were also calculated as integrated predictive indexes, as was the area under the ROC curve. Markers were assessed using a multivariate logistic regression model in a forward stepwise procedure to identify the best combination for predicting brain metastasis. Since ErbB2 was already a known metastasis risk factor, an analysis including ErbB2 as the baseline was also performed, as well as a stratified analysis of each candidate marker within ErbB2-positive and -negative tumors. In all analyses, associations were considered significant when *p* was less than 0.05.

To compare survival times for the control and LND in mice groups, we used the non-parametric Mann-Whitney test and the log-rank test.

The bioluminescence data were transformed using the log (1 + x) function (where x = AvR), in order to obtain a more regular and positive distribution. Subsequently, these data were normalized by subtracting the first observation (day 14) from each of the following observations. The Student's *t* test was used to compare the treatment groups. Survival curves for each treatment were estimated via the Kaplan-Meier method, and the log-rank test was used to assess the significance of differences in both, patients and mice follow-up.

*P*-values lower than 0.05 were considered significant.

## SUPPLEMENTARY DATA, FIGURES AND TABLES


