# Supplementary material for: FN14 and GRP94 expression are prognostic/predictive biomarkers of brain metastasis outcome that open up new therapeutic strategies
Source: Oncotarget. 2015 Oct 19;6(42):44254–73. doi: 10.18632/oncotarget.5471 (PMC4792555; doi:10.18632/oncotarget.5471)
Supplement: Supplementary file 1 [file oncotarget-06-44254-s001.pdf]

SUPPLEMENTARY DATA

Animal models

Athymic Nude-Foxn1nu female mice weighing 22–28 g were purchased from Harlan Laboratories S.A. (Barcelona, Spain) and were housed in the IDIBELL facility in SFP conditions, at 20–24 ºC, 60% relative humidity, and 12–12-hour light-dark periods. All animal-related procedures were performed in accordance with the National Institute of Health Guidelines for the Care and Use of Laboratory Animals, with the approval of the animal care committee.

Xenografts from primary tumors

Samples were collected at Hospital Universitari de Bellvitge (L'Hospitalet de Llobregat, Barcelona, Spain). The study was approved by the Institutional Review Board. Written informed consent was collected from patients. Non-necrotic tissue pieces (2–3 mm^3) from resected ductal breast carcinoma were selected and placed in DMEM (BioWhittaker) supplemented with 10% FBS and penicillin/streptomycin. The xenografts were implanted in animals under isofluorane-induced anesthesia in the intramammary fat path (i.m.f.p). When the i.m.f.p. tumors reached ~1000 mm^3, they were excised, dissected into 2–3 mm^3 cubes and transplanted into additional mice using the same procedure.

Therapeutic protocols

For brain metastasis treatment, we started therapy on day 14 once the mice had recovered from surgery and after checking the success of cell inoculation.

Lenalidomide (LND) obtained from the Celgene Corporation (Summit, NJ) was injected intraperitoneally in DMSO (Sigma-Aldrich) at 50 mg/Kg/day, every day until the end of the experiment. Taxotere (TXT) and NVP-AUY922 (NVP), both from LC Laboratories, were injected intraperitoneally in DMSO at a dosage of 15 mg/Kg/day and 30 mg/Kg/day respectively. Docetaxel was administered every 4 days for 2 weeks and NVP every 2 days for 2 weeks.

Protein expression

Histology and immunohistochemical tumor characterization

The morphology of the engrafted tumors was analyzed by H&E staining in paraffin-embedded sections. Determination of FN14 was performed with anti-FN14 at 1/3000 (Santa Cruz Biotechnology, Santa Cruz, CA) (Tecnique) diluted in Dako Real™ Antibody Diluent Buffer (Dakocytomation): Tris buffer, pH 7.2, 15 mM Na_3N. LSAB+System-HRP (Dakocytomation) was used, including biotinylated anti-rabbit, anti-mouse and anti-goat immunoglobulins in PBS; streptavidin conjugated to HRP in PBS; and liquid 3′3′ diaminobenzidine in chromogen solution.

Western blotting

Cells were lysed in a 1% SDS (v/v) extraction buffer containing an anti-protease cocktail (Roche, Vilvoorde, Belgium). Protein concentrations were determined using the Bradford assay (MicroBCA, Pierce, Belgium). After resolution by SDS-PAGE, electrophoresed proteins were transferred to polyvinylidene fluoride (PVDF) membranes that were blocked and probed with GRP94 (1/1000, Sta Cruz) and the Peroxidase conjugated Antimouse secondary Ab (Pierce, Perbio Science Ltd., Cheshire, U.K). Immunoreactive bands were viewed on a VersaDoc™ (Bio-Rad) Imaging System using the Super Signal west-Pico (Pierce). MWs were established with See Blue Plus2 prestained Standford (Invitrogen, San Diego, CA).

Immune-fluorescence analysis

Cells were analyzed for the expression of FN14. Briefly, 15×10^4 or 60×10^3 cells were seeded in 6 or 24 well-plates containing coverslips. After 24 hours immunofluorescence was performed anti-FN14 primary antibody in PBS1X and SBF 5%, and then fixed with paraformaldehyde at 4% in PBS 1X for 15 min at 4ºC.

For IF analysis, coverslips were mounted on slides using Vectashield (Vector laboratories) with DAPI, which was used for nucleus visualization. Preparations were analyzed with the Olympus BX60 microscope (Olympus Optical Co., Ltd., Tokyo, Japan) and images were taken and analyzed using a digital camera and Spot 4.2 software (Diagnostic Instruments, Inc., Sterling Heights, MI).

Cell viability assay

We used the 3-(4,5-dimethylthiazol-2-yl)-2,5-diphenyltetrazolium bromide assay. The cells were serum starved for 24 h and exposed for a further 72 h to 0–200 ng/mL of TWEAK (PeproTech, PeproTech EC Ltd., London, U.K.).

Statistical analysis

To evaluate the correlation between protein expression and brain metastasis, immunostained samples were graded on a three-category scale (negative, weak positive, and strong positive). The marker was classed as being overexpressed in strong positive samples. The association with brain metastasis for each marker was tested using a two-sided Fisher exact test and summarized by calculating the sensitivity among tumors that developed metastasis, and specificity among tumors

without metastasis, for strong positive values. Positive and negative likelihood ratios were also calculated as integrated predictive indexes, as was the area under the ROC curve. Markers were assessed using a multivariate logistic regression model in a forward stepwise procedure to identify the best combination for predicting brain metastasis. Since ErbB2 was already a known metastasis risk factor, an analysis including ErbB2 as the baseline was also performed, as well as a stratified analysis of each candidate marker within ErbB2-positive and -negative tumors. In all analyses, associations were considered significant when p was less than 0.05.

To compare survival times for the control and LND groups, we used the non-parametric Mann-Whitney test and the log-rank test.

The bioluminescence data were transformed using the $\log(1 + x)$ function (where $x = AvR$), in order to obtain a more regular and positive distribution. Subsequently, these data were normalized by subtracting the first observation (day 14) from each of the following observations. The Student t test was used to compare the treatment groups. Survival curves for each treatment were estimated via the Kaplan-Meier method, and the log-rank test was used to assess the significance of differences.

P-values lower than 0.05 were considered significant.

SUPPLEMENTARY FIGURES AND TABLES

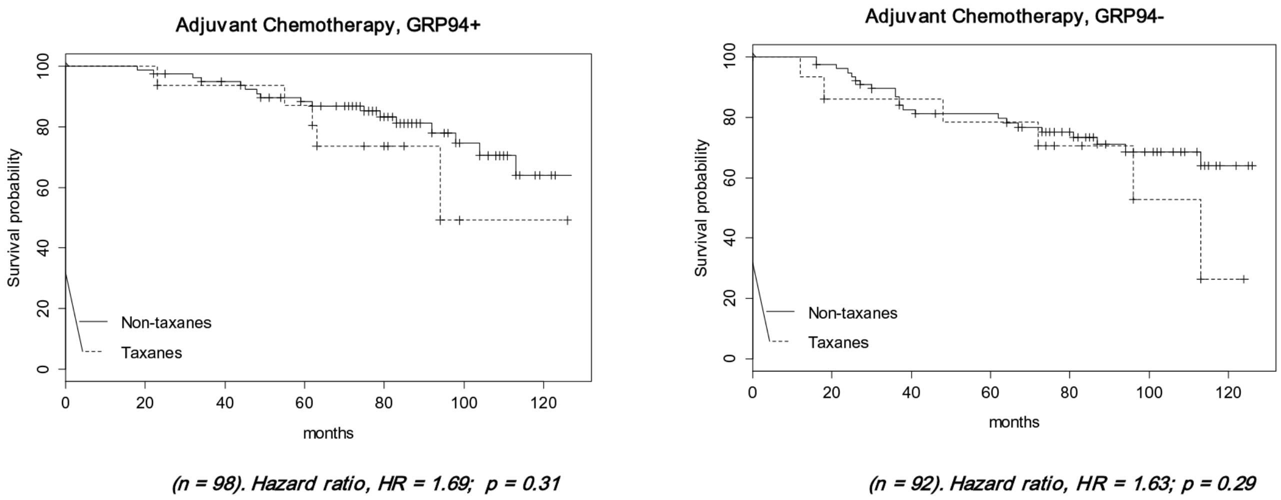

Supplementary Figure S1: Kaplan-Meier survival estimates of overall free survival among patients who received chemoadjuvant therapy, with or without taxanes, according to the GRP94 expression in tumors.

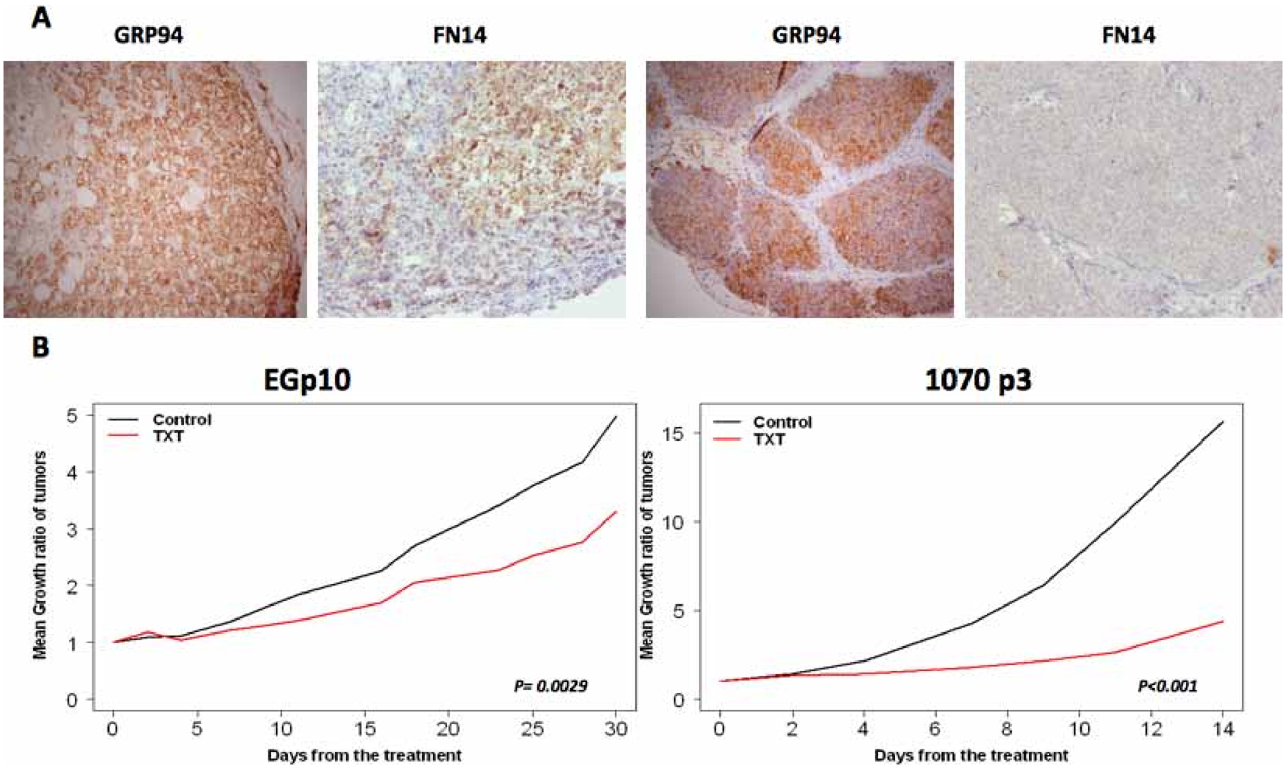

Supplementary Figure S2: Characterization of two different TNBC engrafted in Athymic Nude-Foxn1nu female mice. A. GRP94 FN14 expression by IHC analysis showing TNBC-EG high positivity of both proteins and TNBC-1070 expressing fewer GRP94-positive cells without FN14 expression. **B.** Different slope growth of both subcutaneous engrafts treated with taxotere every 4 days for 2 weeks.

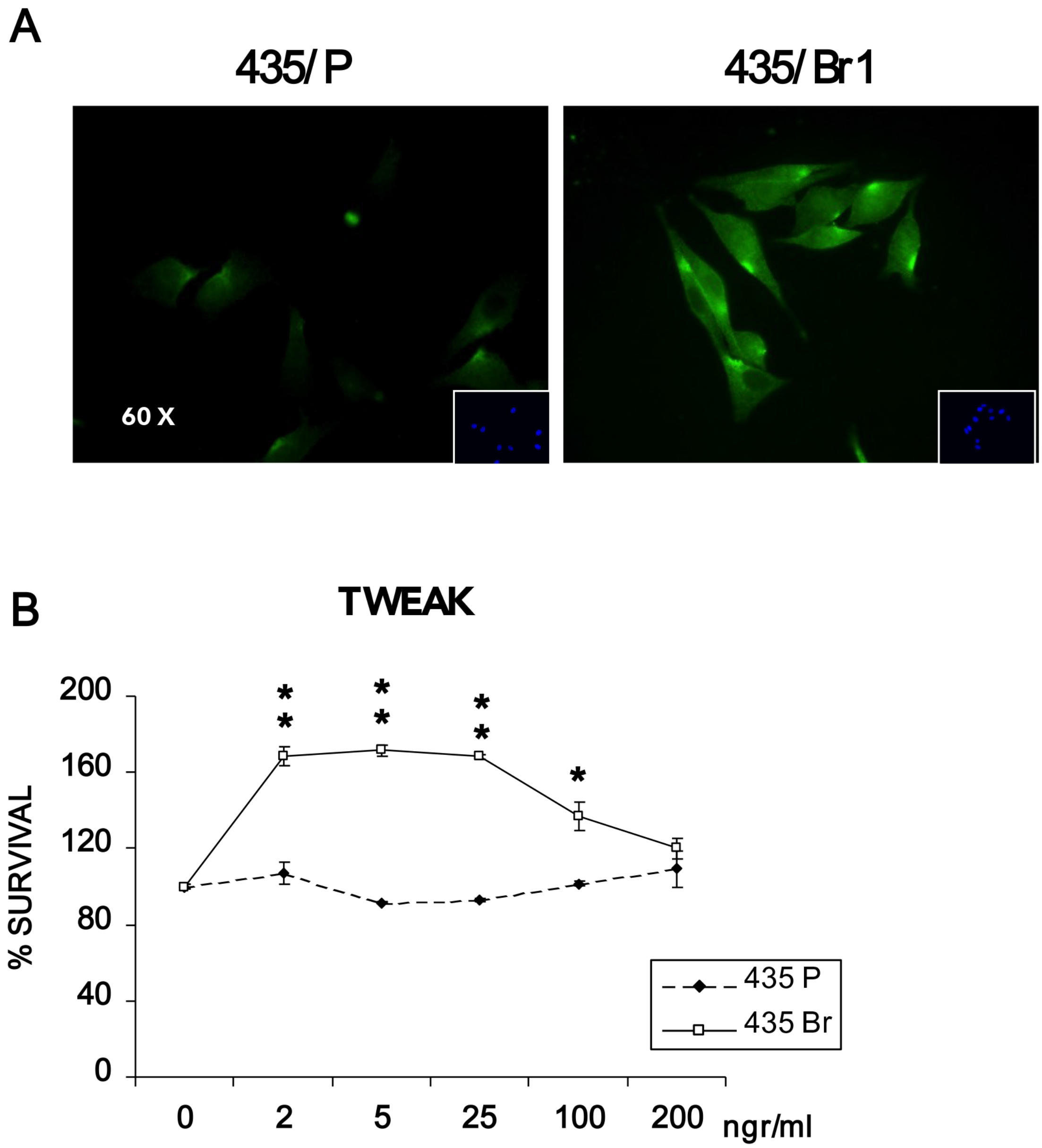

Supplementary Figure S3: MDA-MB 435 parental breast cancer cells (435/P) and the brain metastatic variant 435/Br1 were used for *in vitro* characterization of IMID action. A. Immunofluorescent expression of FN14 (60x) in cells cultured in standard conditions. **B.** 435/P and 435/Br1 cells cultured for 72 hours in the presence of TWEAK at 2–200 ng/mL. The graph is representative of four different experiments that showed a growth advantage of 435/Br1 cells with regard to 435/P when cells were challenged with TWEAK.

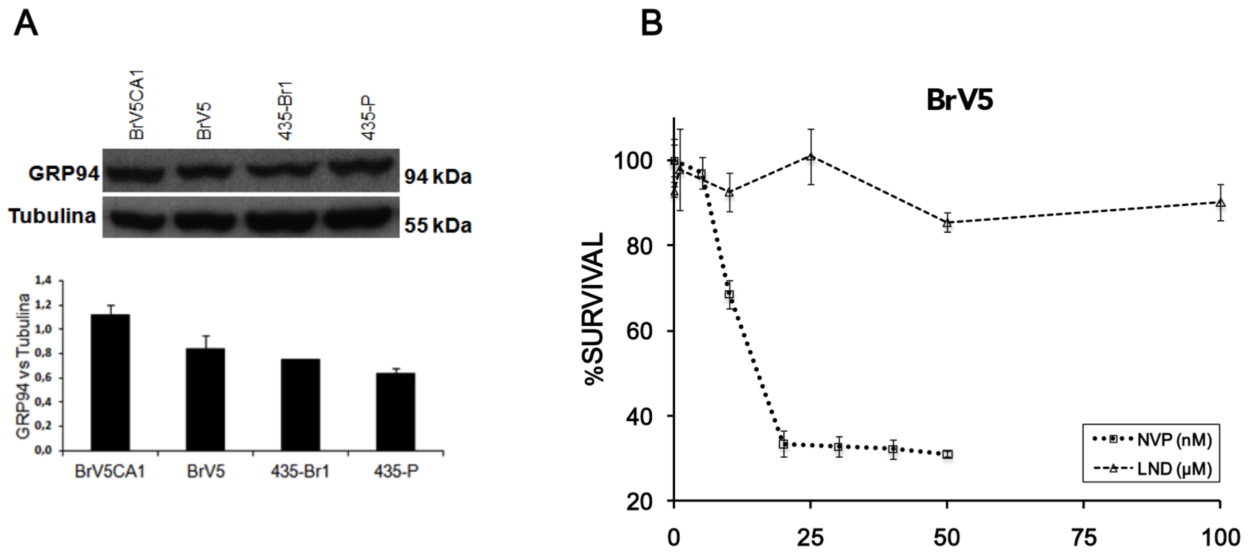

Supplementary Figure S4: Characterization of GRP94 expression in brain metastatic cells. A. Western-blot of 435-P, 435-Br1, BrV5 and BrV5C1 to analyze GRP94 expression (upper panel) and the relative quantification of the protein in cells with regard to the expression of the tubulin (bottom panel. **B.** Survival assay using BrV5 to assess the IC50 of lenalidomide (LND) and the IC50 of heat-shock 90 inhibitor, NVP-AUY922 (right panel). NVP-AUY922 exerted a cytotoxic effect with a IC50 at 50 nM whereas LND did not have a cytotoxic effect on cultured cells.

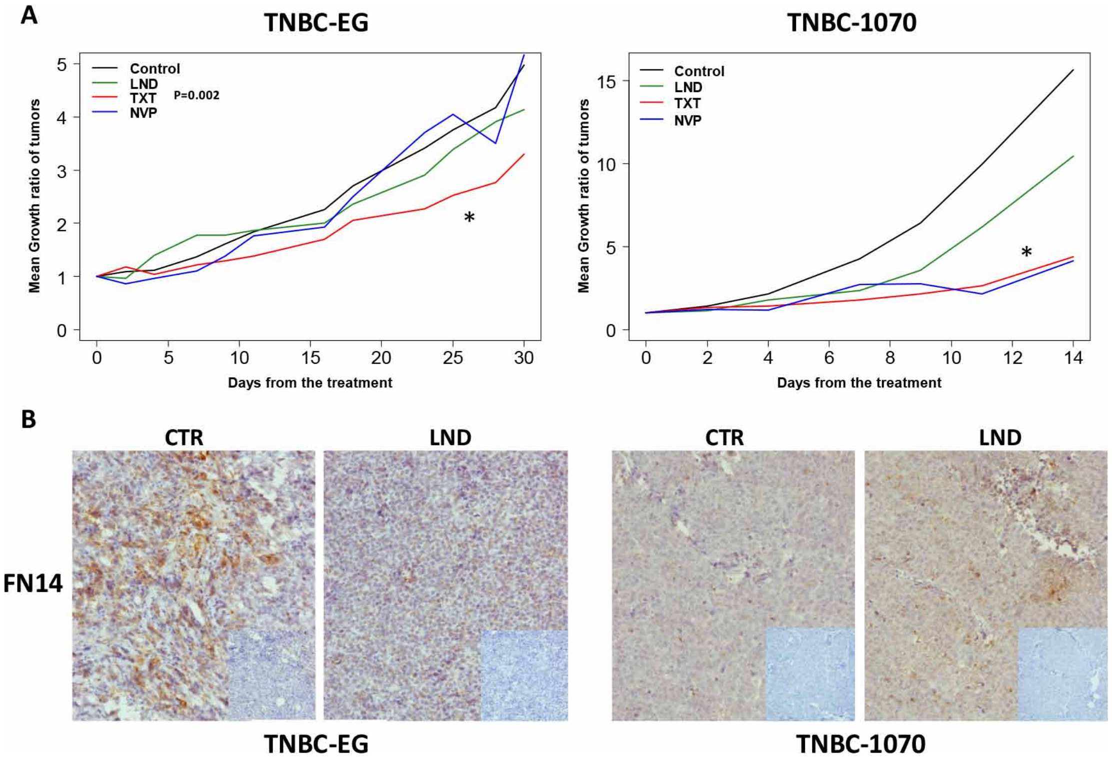

Supplementary Figure S5: The two different TNBC engrafted in Athymic Nude-Foxn1nu female mice were used to check the antitumoral effect of LND with regard to docetaxel and NVP-AUY922. A. The upper panels show TNBC-EG (left) and TNBC-1070 (right) tumor growth slopes when treated with LND (50 mg/Kg/day every day), NVP-AUY922 (30 mg/Kg/day every 2 days for 2 weeks), docetaxel (15 mg/Kg/day every 4 days for 2 weeks) and controls injected with vehicle. TNBC-EG tumors decreased with docetaxel treatment with regard to the control ($p = 0.002$). No effect was observed in mice treated with LND ($p = 0.380$), and the tumor volume decrease was not significant ($p = 0.160$) in NVP-AUY922 treated mice. TNBC-1070 tumors treated with LND had similar volume than controls and differences were not statistically significant ($p = 0.118$). Docetaxel and NVP-AUY922 treatment induced significant reduction of tumor volume with regard to control ($p < 0.001$). **B.** IHC expression of FN14 in tumors treated or not with LND showing the reduced expression in TNBC-EG positive FN14 tumors.

Supplementary Table S1: Brain organ-specific genes/proteins from previously described data based on protein–protein interaction networks analysis (Sanz, et al, 2011 and Sanz et al., 2012)

UP-REGULATED					
**Gen Symbol**	**SwissProt ID**	**Protein Name**	**Function**	***p*-value ***	**Network position (linked to)**
**TRAF2**	**Q96NT2**	**TNF-receptor associated factor 2**	**Signal transduction**	**0.00007**	40 S ribosomal protein s12
**TNFRSF12A**	**Q9NP84**	**Fn14**	**Receptor**	**0.0001**	
**TRA1**	**P14625**	**Glucose regulated protein 94**	**Protein folding**	**0.0009**	
INHA	P05111	Inhibin alpha chain	Signal transduction	**< 0.000001**	
ARFGAP	Q8N6T3	ADP-ribosylation factor GTPase-activating protein 1	Transport	**0.0003**	

(*Continued*)

UP-REGULATED					
**Gen Symbol**	**SwissProt ID**	**Protein Name**	**Function**	***p*-value ***	**Network position (linked to)**
HSPCA	P07900	heat shock 90kDa protein 1, alpha	Chaperone	**0.006**	
ATF6	P18850	ATF-6	ER stress sensors	0.256	
ERN1	O75460	IRE1-alpha	ER stress sensors	0.441	
ERN2	Q76MJ5	IRE1-beta	ER stress sensors	**0.038**	
EIF2AK3	Q9NZJ5	PERK	ER stress sensors	0.155	
DDIT3	P35638	CHOP (GADD135)	UPR pathways	**0.006**	
MAPK8	P45983	JNK1	UPR pathways	**0.009**	
JUN	P05412	c-jun (AP-1)	UPR pathways	**0.006**	
PRKR	P19525	PKR	EIFsK	0.061	
SIRT6	Q8N6T7	Sirtuin 6	Amino acid metabolism	**0.000004**	
ABCA1	O95477	ATP-binding cassette sub-family A member 1	Cholesterol transporter		
AKT/PKB	P31749	RAC-alpha serine/threonine-protein kinase	Protein modification		
SCD-1	O00767	Acyl-CoA desaturase	Lipid desaturation		
LXRA	Q13133	Oxysterols receptor LXR-alpha	Lipid metabolism		
PPARGC1A	Q9UBK2	Peroxisome proliferator-activated receptor gamma coactivator 1-alpha	Transcriptional coactivator of nuclear receptors		
mTOR	P42345	Serine/threonine-protein kinase mTOR	Cell growth modulator		
SRC3	Q9Y6Q9	Nuclear receptor coactivator 3	Nuclear receptor coactivator		
SREBF2	Q12772	Sterol regulatory binding element 1 (SREBP-1)	Lipid/cholesterol metabolism		
SREBF1	P36956	Sterol regulatory binding element 2 (SREBP-2)	Lipid/cholesterol metabolism		
MBTPS1	Q14703	Membrane-bound transcription factor site-1 protease (S1P)	Lipid/cholesterol metabolism		
MBTPS2	O43462	Membrane-bound transcription factor site-2 protease (S2P)	Lipid/cholesterol metabolism		

(*Continued*)

UP-REGULATED					
**Gen Symbol**	**SwissProt ID**	**Protein Name**	**Function**	***p*-value ***	**Network position (linked to)**
ACOT7	O00154	Cytosolic acyl coenzyme A thioester hydrolase (ACOT7)	Fatty-acil CoA binding		
ACACA	Q13085	Acetyl-CoA carboxylase 1 (ACC1)	Fatty acid biosynthesis		
ACACB	O00763	Acetyl-CoA carboxylase 1 (ACC2)	Fatty acid biosynthesis		
FASN	P49327	Fatty acid synthase	Fatty acid biosynthesis		
UCP2	P55851	Mitochondrial uncoupling protein 2	Respiratory electron transport chain		
RPS6KB1	P23443	Ribosomal protein S6 kinase beta-1 (P70S6K1)	ApoptosisCell cycleTranslation regulation		
SLC25A1	P53007	Citrate transport protein	Citrate/malate exchange		
PCYT2	Q99447	Ethanolamine-phosphate cytidyltransferase	phospholipid biosynthesis		
**DOWN-REGULATED**					
RPS23	P62266	40S ribosomal protein S23	Protein biosynthesis	0.000001	40 S ribosomal protein s12
DNM3	Q6P2G1	Dynamin 3	Protein biosynthesis	0.0008	
SERPINB9	P50453	Serpin B9	Signal transduction	0.0007	Tubulin beta-2 chain
CREB1	Q53X93	CAMP responsive element binding protein 1, isoform A	Transcription	0.000005	Vimentin
CREB1	P16220	CAMP responsive element binding protein 1, isoform B	Transcription	0.00005	
AOC3	Q16853	vascular adhesion protein-1	Cell adhesion	0.0004	Glyoxalase I
PDK1	Q15118	Pyruvate dehydrogenase	Inhibits the mitochondrial pyruvate dehydrogenase complex		
C/EBP	P49715	CCAAT/enhancer-binding protein alpha	Transcription regulation		

(*Continued*)

UP-REGULATED					
**Gen Symbol**	**SwissProt ID**	**Protein Name**	**Function**	***p*-value ***	**Network position (linked to)**
SPRC	P09486	Basement-membrane protein 40	Interactions with the extracellular matrix		SPARC
KPCA	P17252	Protein kinase C alpha type	Protein modification		
TP53	P04637	Cellular tumor antigen p53	Growth arrest, apoptosis		
SIRT1	Q96EB6	NAD-dependent deacetylase sirtuin-1	Apoptosis		
EGFR	P00533	Epidermal growth factor receptor	Growth factor		
SUMO3	P55854	Small ubiquitin-related modifier 3	Protein modification		
BTAF1	O14981	TFIID TBP subunit TAF-172(TATA-binding protein-associated factor 172)	Beta tubulin cofactor A		

*Only the root proteins have scored from the previous data. The retrieved interacting proteins were unscored.

Supplementary Table S2: The top 30 genes in the network by GUILD ranking.

GeneSymbol	Rank
TNFRSF12A	1
TRAF2	2
TANK	3
TP53	4
HSP90B1	5
SREBF2	6
ACACB	7
RPS6KB1	8
ACACA	9
PPARGC1A	10
PLSCR4	11
ACOT7	12
MBTPS2	13
MBTPS1	14
INHA	15
SOX30	16
PGRMC1	17
TNFRSF18	18
USP53	19
TRPT1	20
FBXO28	21
FAM120B	22
TNFRSF9	23
TNFSF9	24
EDARADD	25
WDR65	26
PPIL5	27
TNFSF4	28
BANP	29
DLGAP5	30

Supplementary Table S3: GUILD ranks of the BCBrM drug targets and drugs retrieved from DrugBank.

Gene Symbol	Rank	Drug
TNF	194	Thalidomide
PTGS2	246	Thalidomide
FCGR3B	1074	Bevacizumab
NFKB1	1326	Thalidomide
HDAC3	1625	Vorinostat
HDAC1	1859	Vorinostat
HDAC2	2281	Vorinostat
HDAC6	2592	Vorinostat
POLB	2877	Cytarabine
HDAC8	2971	Vorinostat
FCGR2B	3875	Bevacizumab
DHFR	4614	Methotrexate
FCGR2A	5365	Bevacizumab
C1R	5414	Bevacizumab
FCGR1A	5571	Bevacizumab
FGFR2	5800	Thalidomide
C1QA	7462	Bevacizumab
STMN4	7907	Lomustine
C1QC	8290	Bevacizumab
C1QB	8509	Bevacizumab
VEGFA	8659	Bevacizumab
